# Clinical radiation therapy measurements with a new commercial synthetic single crystal diamond detector

**DOI:** 10.1120/jacmp.v15i6.4890

**Published:** 2014-11-08

**Authors:** Wolfram U. Laub, Richard Crilly

**Affiliations:** ^1^ Department of Radiation Medicine Oregon Health & Science University Portland OR USA

**Keywords:** electron dosimetry, photon dosimetry, synthetic single crystal diamond detectors, relative dosimetry, absolute dosimetry

## Abstract

A commercial version of a synthetic single crystal diamond detector (SCDD) in a Schottky diode configuration was recently released as the new type 60019 microDiamond detector (PTW‐Freiburg, Germany). In this study we investigate the dosimetric properties of this detector to independently confirm that findings from the developing group of the SCDDs still hold true for the commercial version of the SCDDs. We further explore if the use of the microDiamond detector can be expanded to high‐energy photon beams of up to 15 MV and to large field measurements. Measurements were performed with an Elekta Synergy linear accelerator delivering 6, 10, and 15 MV X‐rays, as well as 6, 9, 12, 15, and 20 MeV electron beams. The dependence of the microdiamond detector response on absorbed dose after connecting the detector was investigated. Furthermore, the dark current of the diamond detector was observed after irradiation. Results are compared to similar results from measurements with a diamond detector type 60003. Energy dependency was investigated, as well. Photon depth‐dose curves were measured for field sizes 3×3,10×10, and $30\times 30\;{\text{cm}}^{2}$. PDDs were measured with the Semiflex type 31010 detector, microLion type 31018 detector, P Diode type 60016, SRS Diode type 60018, and the microDiamond type 60019 detector (all PTW‐Freiburg). Photon profiles were measured at a depth of 10 cm. Electron depth‐dose curves normalized to the dose maximum were measured with the $14\times 14\;{\text{cm}}^{2}$ electron cone. PDDs were measured with a Markus chamber type 23343, an E Diode type 60017 and the microDiamond type 60019 detector (all PTW‐Freiburg). Profiles were measured with the E Diode and microDiamond at half of $D_{90},D_{90},D_{70}$, and $D_{50}$ depths and for electron cone sizes of $6\times 6\;{\text{cm}}^{2}$, $14\times 14\;{\text{cm}}^{2}$, and $20\times 20\;{\text{cm}}^{2}$. Within a tolerance of 0.5% detector response of the investigated detector was stable without any preirradiation. After preirradition with approximately 250 cGy the detector response was stable within 0.1%. A dark current after irradiation was not observed. The microDiamond detector shows no energy dependence in high energy photon or electron dosimetry. Electron PDD measurements with the E Diode and microDiamond are in good agreement. However, compared to E Diode measurements, dose values in the bremsstrahlungs region are about 0.5% lower when measured with the microDiamond detector. Markus detector measurements agree with E Diode measurements in the bremsstrahlungs region. For depths larger than $d_{\text{max}}$, depth‐dose curves of photon beams measured with the microDiamond detector are in close agreement to those measured with the microLion detector for small fields and with those measured with a Semiflex 0.125 cc ionization chamber for large fields. Differences are in the range of 0.25% and less. For profile measurements, microDiamond detector measurements agree well with microLion and P Diode measurements in the high‐dose region of the profile and the penumbra region. For areas outside the open field, P Diode measurements are about 0.5%–1.0% higher than microDiamond and microLion measurements. Thus it becomes evident that the investigated diamond detector (type 60019) is suitable for a wide range of applications in high‐energy photon and electron dosimetry and is interesting for relative, as well as absolute, dosimetry.

PACS numbers: 00.06, 80.87

## I. INTRODUCTION

Due to their small size and fairly good tissue equivalence, diamond detectors are considered to be of special value in high‐energy photon and electron beam dosimetry. The spatial resolution of this type of detector is comparable to the resolution of commonly used silicon diode detectors^(1)^ whose applicability is limited mainly by their radiation‐induced fading effects and energy dependency to low energy scatter photons. In contrast to this, diamond detectors show high resistance to radiation damage^(2)^ ($0.05\%\cdot {\text{kGy}}^{-1}$). Other advantages are the high sensitivity^1^, ^3^ (about $0.5\;\mu C\cdot {\text{Gy}}^{-1}$) and stability of detector response^(4)^ (relative standard deviation over a 13‐week period of 0.67%), low leakage current^(2)^ ($<5\cdot 10^{-12}\;A$), good temperature stability^(5)^ (better than about $1\%\cdot K^{-1}$), and good time resolution^(6)^ (carrier lifetime is limited to $10^{-8}–10^{-9}\;s$).

In several studies, theoretical aspects of diamond crystals have been discussed.^4^, ^7^, ^8^ Diamond detectors work as solid‐state ionization chambers. When ionizing radiation is absorbed, a temporary change in electrical conductivity is induced by the production of electrons and positive holes which have sufficient energy to freely move through the diamond crystal. The recombination rate in a pure diamond crystal is proportional to the square root of the rate of electron‐hole production and, hence, to dose rate because of the increase in the probability of recombination with the number of vacant holes. Thus pure crystals are unsuitable for dosimetry because of the decrease of charge‐collection efficiency.

In natural diamond crystals impurities are generally present, of which nitrogen is most prominent. Thermal excitation is high enough to ensure that a large fraction of all impurities will be ionized. These ionized impurities can act as traps in the sense that if an electron is captured, it will be immobilized for a period of time which is long enough to prevent that carrier from contributing to the measured pulse.^(9)^

This means that the recombination rate, and hence the efficiency of charge collection, is almost independent of the rate of electron‐hole pair production, resulting in an almost linear increase in detector signal with dose rate. If the concentration of impurities is too high, an insufficient signal will be produced because of the decrease of recombination time. Furthermore, electrons in traps create an electric field which acts in a direction opposite to the applied field produced by external bias voltage. This effect is known as polarization.^5^, ^10^ Thus diamond crystals suitable for radiation dosimetry need a certain concentration of impurities, but too much will cause the diamond to be insensitive and to suffer from polarization effects. Suitable diamond crystals for dosimetry are of the colorless type IIa^11^, ^12^ with a low nitrogen concentration of less than $10^{19}\;{\text{cm}}^{-3}$.

Studies in contact technology have been reported by several authors.^5^, ^6^, ^10^, ^11^ It is necessary to find contact materials which do not lead to an energy dependence of the diamond detector in high‐energy dosimetry. Simultaneously, suitable possibilities of fixing the contacts to the diamond plate have to be found. Polarization can be suppressed by generating space‐charge limited current from the back contact. Therefore, holes have to be injected through the back contact.^5^, ^10^

In summary, diamond selection and contact technology have demonstrated to be of great importance for the properties of diamond detectors. This has resulted in a very limited availability of the initial commercial detector – type 60003 diamond detector (PTW‐Freiburg, Germany). The dependence on natural diamond resulted in a detector series with variations in response reflective of the variation in the composition of diamonds available. In addition, relative dose measurements often have to be corrected to account for dose‐rate dependence of the first commercial detectors.^4^, ^13^

In recent years, Marco Marinelli, Gianluca Verona‐Rinati and their team from the Industrial Engineering Department of Rome Tor Vergata University in Italy have published several studies on the dosimetric characteristics of synthetic single crystal diamond detectors (SCDDs) in a Schottky diode configuration.^14^, ^15^ They tested their SCDDs as radiotherapy dosimeters for small field photon fields of energies up to 10 MV and for electron fields from 6 MeV to 20 MeV. Their studies demonstrate that, as a result of their synthetic production and standardized assembly, SCDDs may offer high reproducibility in their dosimetric properties along with good availability and may, therefore, no longer have the same disadvantages as type 60003 detectors.

A commercial version of such a SCDD was recently released as the new type 60019 microDiamond detector (PTW‐Freiburg, Germany). In this study we investigate the dosimetric properties of this detector to independently confirm that findings from the developing group of the SCDDs still hold true for the commercial version of the SCDDs. This paper explores whether the microDiamond detector can be expanded to higher energy photon beams of up to 15 MV and to large field measurements.

## II. MATERIALS AND METHODS

Measurements were performed with an Elekta Synergy linear accelerator (Elekta, Stockholm, Sweden) delivering 6, 10, and 15 MV X‐rays, as well as 6, 9, 12, 15, and 20 MeV electron beams.

The radiation‐sensitive region of the investigated microDiamond detector type 60019 (PTW Freiburg) is a synthetic single crystal diamond crystal with a disk‐shaped nominal sensitive volume of $0.004\;{\text{mm}}^{3}$. This diamond plate is fixed in a polystyrene and epoxy housing of diameter 7.0 mm, the surface of the crystal lies 1.0 mm (1.275 mm for the used prototype) below the top of the housing. The unbiased diamond detector was connected to a Unidos E Universal Dosimeter for response measurements and to a Tandem Dual‐channel electometer for scanning measurements (PTW‐Freiburg). Dose measurements were carried out in a MP3‐M water phantom (PTW‐Freiburg). The diamond detector was positioned at the central beam axis of a high‐energy photon or electron beam, entering the surface of the water phantom perpendicularly. To follow vendor guidelines a preirradiation of the detector with a dose of about 8 Gy was delivered to settle the response of the diamond at a stable level.

The dependence of the microdiamond detector response on absorbed dose after connecting the detector was investigated. For this purpose, the diamond detector was positioned at a depth of 10 cm in a $10\times 10\;{\text{cm}}^{2}$ photon field and repeatedly irradiated with 50 MU of a 6 MV photon beam (34 cGy). Furthermore, the dark current of the diamond detector was observed after irradiation. Results are compared to similar results from earlier measurements made with a diamond detector type 60003.

To investigate energy dependency of the microdiamond detector response, the diamond detector was positioned at a depth of 10 cm in a $10\times 10\;{\text{cm}}^{2}$ field for and tested for a number of photon energies. Similar tests were done with electron beam measurements at reference depths with a $10\times 10\;{\text{cm}}^{2}$ cone. After preirradiation, the microDiamond was then irradiated with 100 cGy.

Photon depth‐dose curves normalized to the dose maximum (PDDs) were measured for all available photon energies at a source‐to‐surface distance (SSD) of 100 cm and for field sizes 3×3,10×10, and $30\times 30\;{\text{cm}}^{2}$. PDDs were measured with the Semiflex type 31010 detector (10×10 and $30\times 30\;{\text{cm}}^{2}$), microLion type 31018 detector (3×3 and $10\times 10\;{\text{cm}}^{2}$), P Diode type 60016, SRS Diode type 60018 (3×3 and $10\times 10\;{\text{cm}}^{2}$), and the microDiamond type 60019 detector (all PTW‐Freiburg). Photon profiles were measured with the same detectors at a depth of 10 cm.

Electron depth‐dose curves normalized to the dose maximum were measured for all available electron energies with the $14\times 14\;{\text{cm}}^{2}$ electron cone and for 6 MeV and 20 MeV, in addition, with the $6\times 6\;{\text{cm}}^{2}$ and $20\times 20\;{\text{cm}}^{2}$ cones. PDDs were measured with a Markus chamber type 23343, an E‐Diode type 60017, and the microDiamond type 60019 detector (all PTW‐Freiburg). Profiles were measured with the same detectors at half of $D_{90},D_{90},D_{70}$ and $D_{50}$ depths.

## III. RESULTS & DISCUSSION

### A. Diamond detector response

**Figure 1:**
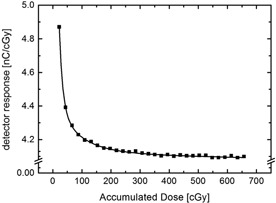
Response of the old diamond detector type 60003 versus absorbed dose after the detector had not been used for two hrs. The squares (■) represent the average response during irradiation steps of 22 cGy with a 6 MV photon beam at a dose rate of 290 c; the straight line (—) represents the best exponential fit.

**Figure 2:**
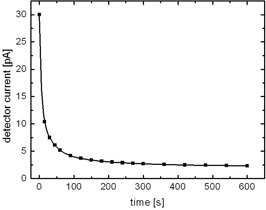
Dark current of old diamond detector type 60003 after irradiation with a 6 MV photon beam vs. time. The best exponential fit is plotted.

The detector response of the earlier commercial diamond detector (type 60003) is found to initially decrease significantly with absorbed dose. This can be attributed to the increase of the described polarization effect as traps fill.^(10)^ If an equilibrium trap population is reached, the response of the detector settles at a stable level. After an exponential falloff in response of approximately 19%, a stable response of about $4.1\cdot 10^{-7}C\cdot {\text{Gy}}^{-1}$ is attained when a dose greater than about 5 Gy has been absorbed (Fig. 1). This asymptotic effect was found to occur at the initiation of every irradiation separated by as little as a few hours. In this investigation, a change in response could be established if irradiation was interrupted for a few minutes, provided that the high voltage of +100V was switched on during the break as well as during irradiation. This finding is related to the behavior of the dark current after irradiation (Fig. 2). Immediately after irradiation, the dark current was measured to approximately 30 pA. The space charge generated by trapped electrons inside the diamond crystal initially makes a contribution to dark current. However, after about 5 min, the dark current stabilizes at approximately 2.5 pA. This indicates that most traps have been emptied. Consequently, the diamond detector type 60003 has to be preirradiated until an equilibrium in trap population is reached and thus a stable detector current is reached. If the high voltage is switched off after irradiation, the dark current decreases significantly more slowly. Thus, a change in detector response can be noticed only after a prolonged period of time.

**Figure 3:**
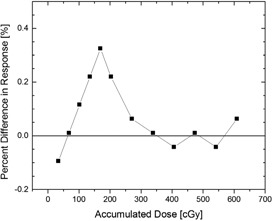
Percent difference in microDiamond detector response from average response (%) after an accumulated dose of 250 cGy has been delivered to the detector. After 250–300 cGy of accumulated dose, the response is stable within 0.1%.

Behavior of a microDiamond detector type 60019 is very different. This can be explained with the Schottky diode configuration of the detector and the fact that no voltage is applied to the microDiamond detector during measurements.^(14)^ Within a tolerance of 0.5%, detector response of the investigated detector was stable without any preirradiation. After preirradition with approximately 250 cGy, the detector response was stable within 0.1% (Fig. 3). A dark current after irradiation was not observed. These findings are similar to those from Ciancaglioni et al.,^(14)^ which showed that detector signal stability within 0.5% is achieved after a preirradiation dose of approximately 60 cGy. Based on these results, the vendor recommendation to preirradiate the microDiamond detector with 800 cGy before seems very conservative. Preirradiating microDiamond detectors with approximately 300 cGy would be sufficient for the investigated detector, but it is of course possible that other type 60019 detectors might need to be preirradiated longer before settling for a stable signal.

### B. Energy dependence

**Figure 4:**
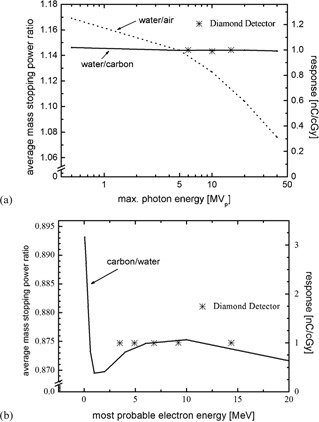
Response of the microDiamond detector for photon (a) and electron (b) beams of different energies. For reference, average mass stopping power ratios water/air (dashed line) and water/carbon (solid line) are displayed.

The response of the diamond detector was determined for photon beams of different energy. The detector response does not change significantly (<1%) and is well within the measurement uncertainty of an estimated 2%; therefore, it can be said that the diamond detector shows no energy dependence within the covered energy range of 6–15 MV photon beams (Fig. 4(a)). Although this is expected due to the near tissue equivalence of carbon, the contact material of the detector and the PTW‐type housing might introduce a certain energy dependence.

For electron beams, the response of the diamond detector was determined for different energies. The response does not change significantly (<1%) and is well within measurement uncertainty. It can be said that the diamond detector shows no energy dependence within the covered energy range from 6–20 MeV electrons beams (Fig. 4(b)). The energy dependence is less, as one would expect when looking at mass stopping power ratio values for carbon and water in monoenergetic electron beams. It was noted that the average detector response for electron beams is less than 0.5% different compared to the average detector response for photon beams. These findings agree to those of prototype SCDDs with different detector housing.^(15)^

### C. Dose distributions of electron beams

Depth‐dose curves of electron beams measured with the diamond detector look rather different from depth‐ionization curves measured with Markus chamber. Of course, relative ionization values measured with the Markus chamber must be corrected with the water to air stopping power ratio $4.1\cdot 10^{-7}C\cdot {\text{Gy}}^{-1}$, which decreases with increasing energy. Because of the decrease of the electron energy in water, $4.1\cdot 10^{-7}C\cdot {\text{Gy}}^{-1}$ is dependent on the initial energy of the electron beam and on the water depth z. The practical range $R_{p}$ of electrons with the most probable energy at water surface $E_{p,0}$ can be calculated for $1\text{MeV}\leq E_{p,0}\leq 5\text{MeV}$ by the empirical range expression:^(16)^

(1) $$R_{p}=-0.11+0.505\cdot E_{p,0}-3\cdot 10^{-4}\cdot E^{2}{\;}_{p,0}$$

$({S_{W/a})}_{E}$ is approximately given by

(2) $${\left(S_{W/a}\right)}_{E}=\frac{1}{3.871+0.549\cdot R_{r}}+0.867$$

where the residual range (in cm) $R_{r}$ results from the difference $R_{r}=R_{p}–z$. This procedure to calculate the energy correction factor $({S_{W/a})}_{E}$ for each initial energy and water depth z is only an approximate correction of the response of ionization chambers. Further, the diamond detector has a distinctly better spatial resolution than a Markus chamber. Therefore, in regions of high‐dose gradients of electron depth‐dose curves, distinctions appear due to the different spatial averaging of both detectors. Another effect contributes to the remaining difference in the measurements. Already before the electrons reach their maximum range in water, they emit bremsstrahlung. However, the energy correction, which is adequate solely for electrons, is carried out without consideration of the share of the measured dose caused by bremsstrahlung.

**Figure 5:**
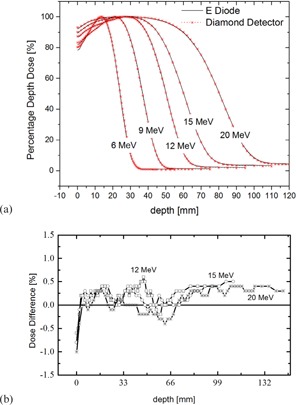
Comparison between electron percent depth‐dose curves as measured with an E Diode and diamond detector (a). Other than small differences in the high‐dose gradient areas of the PDDs, a difference of about 0.5% between the two measurements is noticeable in the bremsstrahlungs region (b).

**Figure 6:**
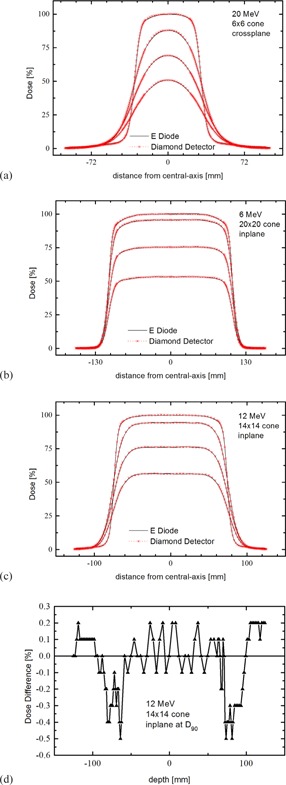
Profile measurement for 20 (a), 6 (b), and 12 (c) MeV electron beams in 6×6,20×20, and 14×14 cones. Measurements with an E Diode and diamond detector are in excellent agreement for in‐plane and cross‐plane profiles. Dose differences are in the order of 0.5% and less, as the difference plot for 12 MeV profile measurements (d) shows.

To better appreciate these effects, it was felt that it would be useful to compare measurements between E Diode and microDiamond detectors (Figs. 5 and 6). E Diodes, as well as diamond detectors, do not require the above correction and directly measure dose (dose to silicon and dose to carbon, respectively, which are both close to dose to water) rather than ionization. They also have a similar high spatial resolution.

Profile and PDD measurements with the E Diode and microDiamond are in good agreement and even in high‐gradient areas only show differences of 1% and less. However, compared to E Diode measurements, dose values in the bremsstrahlungs region of PDDs are about 0.5% lower when measured with the microDiamond detector. Markus detector measurements agree with E Diode measurements in the bremsstrahlungs region. A similar effect, but to a lesser extent, can be observed in the publication from Venanzio et al.,^(15)^ though it is not discussed in their paper. One explanation for this behavior is that, where diamond detectors show very similar response to electron and photon radiation, both the E Diode as well as the Markus detector might overrespond to the bremsstrahlungs radiation in the electron beam, therefore slightly overestimating the percent dose in the bremsstrahlungs region. This will need to be investigated further but, at the same time, the difference is small and clinically of no significant importance.

### D. Dose distributions of photon beams

**Figure 7:**
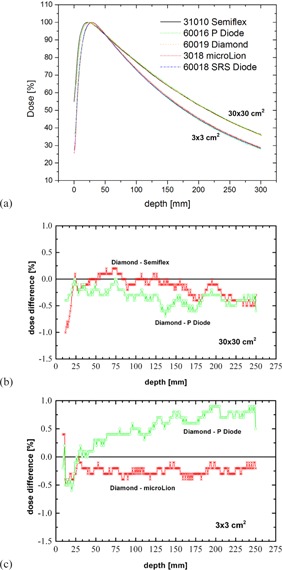
15 MV PDD measurements (a) for field sizes $3\times 3\;{\text{cm}}^{2}$ and $30\times 30\;{\text{cm}}^{2}$ with different detectors. For the large field (b), the difference between microDiamond and Semiflex detector and between microDiamond and P Diode is displayed. For small fields (c), the difference between microDiamond and microLion and between microDiamond and P Diode is shown. In both cases, the difference between the microDiamond and P Diode is about twice as much as the difference between the microDiamond detector and ionization chamber.

For depths larger than $d_{\text{max}}$, depth‐dose curves of photon beams (6 MV, 10 MV, and 15 MV) measured with the microDiamond detector are in close agreement to those measured with the microLion detector for small fields ($3\times 3\;{\text{cm}}^{2}$) and with those measured with a Semiflex 0.125 cc ionization chamber for large fields ($30\times 30\;{\text{cm}}^{2}$). Differences are in the range of 0.25% and less. In comparison to this, differences between the microDiamond detector and P Diode measurements are larger and between 0.5% to 1.0% (Fig. 7). In the buildup region, microDiamond detector measurements agree well with high‐resolution detectors. Thus, the microDiamond detector can be considered suitable for small and large field photon PDD measurements and even produces results superior to P Diode measurements.

**Figure 8:**
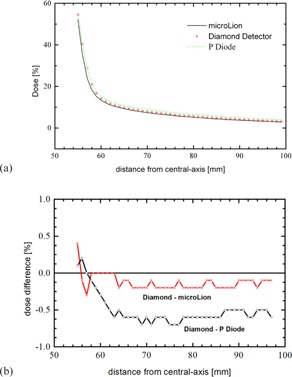
Low‐dose area of a $10\times 10\;{\text{cm}}^{2}\;6$ MV photon field at 10 cm depth as measured with a P Diode, microLion, and microDiamond detector (a). It can be seen that the P Diode overresponds to low‐energy scatter photons in this region of the profile (b). Profiles were all normalized to central axis and good agreement was found for all three detectors in the high‐dose region and penumbra region of the profile.

For in‐ and cross‐plane profile measurements, microDiamond detector measurements agree well with microLion and P Diode measurements in the high‐dose region of the profile and in the penumbra region. 80%/20% penumbra values for a 6 MV $10\times 10\;{\text{cm}}^{2}$ profile in in‐plane direction, for example, were 5.6 mm with a P Diode, 5.69 mm with the microDiamond detector, and 6.16 with the microLion detector. For low‐dose areas outside the open field, P Diode measurements are about 0.5%–1.0% higher than microDiamond and microLion measurements, whereas microDiamond and microLion measurements agree within 0.25% (Fig. 8). Because of its energy dependency, the P Diode over‐responds to low energy scatter photons in this region.

## IV. CONCLUSIONS

Our results show that the microDiamond detector type 60019 is a useful measuring device in high‐energy photon and electron dosimetry. A preirradiation of about 3 Gy was sufficient for the investigated detector to lower the stability of response from 0.5% to 0.1%. In contrast to an ionization chamber, the investigated microDiamond detector shows no energy dependence in high‐energy photon and electron dosimetry.

The diamond detector was found to have a superior spatial resolution and at the same time produced reliable measurements for small and large field photon PDD and profile measurements, as well as for electron PDD and profile measurements. The applications that most benefit from this type of detector might be the small or spatially complex dosimetry associated with brachytherapy or stereotactic radiotherapy, particularly in inhomogeneous media. Further, it appears evident that the investigated microDiamond detector is suitable for a wide range of applications in dosimetry, including absolute dosimetry, given proper calibration procedures are developed. As a result, this detector could easily replace E Diode, P Diode, and microLion detectors and become the universal detector for the commissioning of linear accelerators or other ongoing QA measurements on linear accelerators.

## ACKNOWLEDGMENTS

The authors would like to thank PTW‐Freiburg for providing us with a prototype of their new commercial microDiamond detector type 60019 for this study.
